# 1833. Infectious Diseases in Patients Experiencing Homelessness in Detroit, MI Over a Three-Year Period (2020-2023)

**DOI:** 10.1093/ofid/ofad500.1662

**Published:** 2023-11-27

**Authors:** Andrew J Failla, Marcus Zervos

**Affiliations:** Henry Ford Hospital, Detroit, Michigan; Henry Ford Hospital, Detroit, Michigan

## Abstract

**Background:**

The homeless population has many health challenges. In Detroit there are estimated 8000 people experiencing homelessness, 62% with a disabling condition, and an estimated readmission rate 4x greater than non homeless (Homeless Action Network Detroit). Coordinating care presents unique challenges. It is imperative to examine infections over the long term to identify areas for care improvement, education, infection prevention and better access. We show an infection profile in patients experiencing homelessness from admissions over 3 years at a large hospital in Detroit. This will bring to light the needs of this population and foster delivery of equitable care to the classically underserved.

**Methods:**

This is a retrospective cohort study done over a 3 year period from 2020-2023 in homeless patients hospitalized at a 850 bed urban hospital. In total we were able to identify 200 patients, and 31 patients had information collected from the EMR.

**Results:**

Patients were mostly Black (83%) males (77%). 48% had primary care providers. 87% had history of substance abuse (35% endorsing IV). Of the 31 patients, all had an infection during the study period. 16% with diagnosis of HIV, and 42% had Hep C. Most common infections were as follows: soft tissue (51.6%), pneumonia (29%), urinary tract (16%). Most common organism in culture was Staph aureus (42%; 46% MRSA), Strep (13%) and E coli (13%). Of gram negatives, 20% were ESBL and 20% AmpC. 35% had a surgical procedure while inpatient. The most common antibiotics were vancomycin (35%) and cephalosporins (32%). Treatment duration was usually 7-10 days (29%). 61.3% completed treatment, 16% had recurrent infection, and 9.6% lost to follow up. 55% received ID consult, but only 9.6% followed up in clinic. 19.3% saw a street medicine organization. Readmission was low (9.6%), although 19.3% had an ER visit post discharge.

**Conclusion:**

This preliminary study establishes a profile of infections in those experiencing homelessness in Detroit. This data will help educate providers, allow equitable and targeted care to be delivered, and lead to intervention options that improve access to care. In the future we plan to create useful tools such as an antibiogram specific to this population that can help community health organizations impact care delivered at initial contact.

**Disclosures:**

**Marcus Zervos, MD**, Contrafect: Advisor/Consultant|GSK: Grant/Research Support|Johnson and Johnson: Grant/Research Support|Pfizer: Grant/Research Support

